# Multicenter Thai university-based surgical ICU study (Thai-SICU study): adverse events and outcome in the SICU

**DOI:** 10.1186/cc14598

**Published:** 2015-03-16

**Authors:** S Kongsayreepong, K Chittawatanarat

**Affiliations:** 1 Siriraj Hospital, Mahidol University, Bangkok, Thailand

## Introduction

The aim of this Thai-SICU was to study the incidence and outcome of adverse events in nine SICU university-based hospitals in Thailand.

## Methods

This multicenter prospective observational study was done in >18 years old, admission >6 hours surgical patients admitted to the large, postgraduate medical training university-based SICU during April 2011 to January 2012. Patient data were divided into three main phases as admission, discharge data and daily CRFs during the ICU stay. The patients were followed until they were discharged from the ICU or up to 28 days of their ICU admission and up to 28 days following discharge from the ICU if they survived.

## Results

Following a 19.7-month recruitment period, a total of 4,654 patients (17,579 ICU-days) were included in the analysis process. Admitted patients had the median age of 64 years. Most of the patients were admitted directly from the OR for postoperative monitoring with median APACHE II score 10, 23% were admitted with priority I who needed aggressive hemodynamic resuscitation and respiratory support. ICU mortality and 28-day mortality were 9.61% and 13.80%. Each day of ICU increment was associated with a 1.38-day increase of hospital stay (95% CI, 1.24 to 1.53; *P *< 0.01). On the surveillance periods, the six most common adverse events were sepsis (19.5%), AKI (16.9%), new cardiac arrhythmias (6.17%), respiratory failure (5.83%), cardiac arrest (4.86%) and delirium (3.5%) respectively. The other events including reintubation within 72 hours, intraabdominal hypertension, acute MI, unplanned extubation, upper GI hemorrhage, pneumohemothorax, seizure, drug error and pulmonary aspiration were <3% each. The risk of adverse events on 28-day mortality were significant on cardiac arrest (RR = 9.45; *P *< 0.01), ARDS (RR = 4.58; *P *< 0.01), AKI (RR = 4.18; *P *< 0.01), sepsis (RR = 3.62; *P *< 0.01), iatrogenic pneumohemothorax (RR = 3.23; *P *< 0.01), seizure (RR = 3.12; *P *< 0.01), upper GI hemorrhage (RR = 2.97; *P *< 0.01), cardiac arrhythmia (RR = 2.91; *P *< 0.01), ALI (RR = 2.71; *P *< 0.01), delirium (RR = 2.13; *P *< 0.01), MI (RR = 2.12; *P *< 0.01), unplanned extubation (RR = 2.06; *P *< 0.01), abdominal hypertension (RR = 1.75; *P *< 0.01) and reintubation within 72 hours (RR = 1.51; *P *= 0.02).

## Conclusion

This is the largest systemic surveillance observation in the SICU. The study results are the reference for future research and also provide information for patient and relative advice when confronted with adverse events during SICU admission.

